# The pharmacokinetics and drug-drug interactions of ivermectin in *Aedes aegypti* mosquitoes

**DOI:** 10.1371/journal.ppat.1009382

**Published:** 2021-03-17

**Authors:** Urs Duthaler, Michael Weber, Lorenz Hofer, Carlos Chaccour, Marta Maia, Pie Müller, Stephan Krähenbühl, Felix Hammann

**Affiliations:** 1 Division of Clinical Pharmacology & Toxicology, Department of Biomedicine, University and University Hospital Basel, Basel, Switzerland; 2 Division of Clinical Pharmacology & Toxicology, Department of Pharmaceutical Sciences, University of Basel, Basel, Switzerland; 3 Swiss Tropical and Public Health institute, Basel, Switzerland; 4 University of Basel, Basel, Switzerland; 5 ISGlobal, Hospital Clínic—Universitat de Barcelona, Barcelona, Spain; 6 Facultad de Medicina, Universidad de Navarra, Pamplona, Spain; 7 Ifakara Health Institute, Ifakara, United Republic of Tanzania; 8 Kenyan Medical Research Institute, Wellcome Trust Research Programme, Department of Biosciences, Kilifi, Kenya; 9 University of Oxford, Nuffield Department of Medicine, Centre for Global Health and Tropical Medicine, Oxford, United Kingdom; 10 Clinical Pharmacology and Toxicology, Department of General Internal Medicine, Inselspital, Bern University Hospital, University of Bern, Switzerland; University of Pennsylvania, UNITED STATES

## Abstract

Mosquitoes are vectors of major diseases such as dengue fever and malaria. Mass drug administration of endectocides to humans and livestock is a promising complementary approach to current insecticide-based vector control measures. The aim of this study was to establish an insect model for pharmacokinetic and drug-drug interaction studies to develop sustainable endectocides for vector control. Female *Aedes aegypti* mosquitoes were fed with human blood containing either ivermectin alone or ivermectin in combination with ketoconazole, rifampicin, ritonavir, or piperonyl butoxide. Drug concentrations were quantified by LC-MS/MS at selected time points post-feeding. Primary pharmacokinetic parameters and extent of drug-drug interactions were calculated by pharmacometric modelling. Lastly, the drug effect of the treatments was examined. The mosquitoes could be dosed with a high precision (%CV: ≤13.4%) over a range of 0.01–1 μg/ml ivermectin without showing saturation (R^2^: 0.99). The kinetics of ivermectin were characterised by an initial lag phase of 18.5 h (CI_90%_: 17.0–19.8 h) followed by a slow zero-order elimination rate of 5.5 pg/h (CI_90%_: 5.1–5.9 pg/h). By contrast, ketoconazole, ritonavir, and piperonyl butoxide were immediately excreted following first order elimination, whereas rifampicin accumulated over days in the mosquitoes. Ritonavir increased the lag phase of ivermectin by 11.4 h (CI_90%_: 8.7–14.2 h) resulting in an increased exposure (+29%) and an enhanced mosquitocidal effect. In summary, this study shows that the pharmacokinetics of drugs can be investigated and modulated in an *Ae*. *aegypti* animal model. This may help in the development of novel vector-control interventions and further our understanding of toxicology in arthropods.

## Author summary

Mosquitoes are responsible for the transmission of pathogens, which cause diseases that are of major health significance such as dengue fever and malaria. Preventive strategies involving the use of insecticides, however, have led to the emergence of resistant mosquitoes. Consequently, development of complementary approaches is urgently needed to stop the spread of these pathogens. Our study reports on a pioneering approach to investigate how well drugs are taken up by the mosquitoes and how long they reside in their body. We focused on ivermectin, which is toxic for mosquitoes, and several drugs that interfere with drug metabolising enzymes. We demonstrated that the exposure of drugs can be precisely determined in individual mosquitoes and that drugs interact with each other in the same way as observed in vertebrates. In this regard, we were able to increase the exposure and mosquito toxicity of ivermectin by co-administering ritonavir, a broad-spectrum inhibitor of drug metabolising enzymes. This study establishes *Aedes* mosquitoes as a new model organism for pharmacokinetic studies. It opens the door for the investigation of novel insecticide strategies and optimisation of lead compounds against mosquitoes.

## Introduction

The World Health Organization (WHO) estimates vector-borne diseases to account for more than 17% of all infectious diseases [1]. Arthropods such as mosquitoes, sand flies, and ticks are arguably the most important disease vectors. Mosquitoes in particular transmit diseases that are a major threat to global health. In 2018 alone, *Anopheles* mosquitoes caused an estimated 228 million malaria cases resulting in 405,000 deaths [2]. With the global spread of the highly efficient urban vector *Aedes aegypti*, increasing international travel, and climate change, arthropod-borne viruses (arboviruses) including dengue, Zika, yellow fever and chikungunya are (re-)emerging [3]. Besides malaria, dengue fever has the highest disease burden globally with an estimated annual incidence of 390 million and with about half the world’s population being at risk [4,5].

Although efforts are underway to develop novel drugs and vaccines against mosquito-borne pathogens, drug resistance and an impervious protective immunity are major obstacles towards eradicating these diseases. In addition, many vector control interventions such as indoor residual spraying and insecticide-treated nets seem to be reaching an efficacy plateau as mosquitoes adapt their behaviour or develop insecticide resistance [6,7].

A novel potential complementary approach to vector control is mass administration of endectocidal drugs to humans and livestock. Modelling and preliminary clinical trials using the antiparasitic drug ivermectin have shown that this strategy could effectively reduce malaria transmission by targeting the mosquito population [8,9]. Ivermectin is lethal for *Anopheles* mosquitoes already in the low nanomolar range, while sub-lethal concentrations lead to mosquitoes to producing fewer offspring [10–12]. Ivermectin is less effective against *Aedes* mosquitoes on the other hand: while a single oral dose of 150 μg/kg yields host blood concentrations lethal to *Anopheles* mosquitoes, it would only reach within 10% of the LD_50_ for similar effects against *Ae*. *aegypti* [13]. Thus, ivermectin is not considered a candidate endectocide for arboviral disease control [14]. Nevertheless, *Aedes* mosquitoes serve as a good model system to study the pharmacokinetics (PK) and drug-drug interactions of endectocides, because laboratory strains are simple to rear and readily membrane-feed on human blood. Moreover, *Ae*. *aegypti’s* tolerance to high ivermectin concentrations allows for drug quantification in single mosquitoes.

Effective control with mass drug administration requires a large proportion of the human population to receive the drug. The intake of co-medications for concomitant disorders may impact the efficacy and safety of the intervention through drug-drug interactions in the host and the mosquito. Tuberculosis and HIV/AIDS present the greatest challenge, as they are widely co-endemic with mosquito-borne diseases. The growing availability of medical care results in a considerable share of the human target populations receiving medication that may lead to relevant drug-drug interactions [15], but potentially also in the mosquito. Rifampicin, one of the mainstays of tuberculosis treatment, is also one of the strongest activators of the nuclear pregnane X receptor (PXR). PXR is associated with a wide variety of drug-metabolising enzymes and drug transporters such as cytochrome P450 3A4 (CYP3A4) and p-glycoprotein (Pgp), both of which ivermectin interacts with. HIV anti-retroviral therapy may consist of broad CYP inhibitors and/or inducers such as the protease inhibitor ritonavir. Ivermectin itself is metabolised by CYP3A4 by which it is demethylated at the disaccharide substituent and/or hydroxylated at several positions of the aglycon [16]. It is unknown to what extent the metabolites contribute to the activity of ivermectin, thus the impact of CYP induction or inhibition is difficult to estimate [17]. A drug interaction study in pigs showed, however, that co-administration of ivermectin and ketoconazole, a strong inhibitor of CYP3A4, increased ivermectin´s activity against *Anopheles gambiae* mosquitoes [18]. The study concluded that co-medications may affect the PK of endectocides within humans, while drug-drug interactions may be equally possible in the arthropod vector. Overall, interactions at both sites might compromise the success of mass endectocide administration.

The aim of the present study was to investigate the PK of ivermectin and its interaction with co-administered medications in *Ae*. *aegypti* mosquitoes. Ivermectin was administered by membrane blood feeding either alone or in combination with ketoconazole, ritonavir, rifampicin, or piperonyl butoxide, an unselective inhibitor of CYPs in insects [19]. While conventional pharmacokinetic modelling describes concentration profiles in central or effect compartments, measuring systemic concentrations in mosquito tissues is impossible from a technical and anatomical standpoint. As a result, only whole specimens can be analysed at the scale required for pharmacokinetic studies. Therefore, a sparse-sampling population modelling approach based on the time course of whole-body amounts in individual mosquitoes was implemented. Concentration-time profiles were created based on drug level measurements of single mosquitoes, whereas the primary pharmacokinetic parameters and the effect of drug-drug interactions on model parameters such as clearance were assessed by pharmacometric modelling.

## Results

### Raw data of the main and supporting information figures are given in S1 Data

#### Mosquito dosing experiments

**Fig 1:**
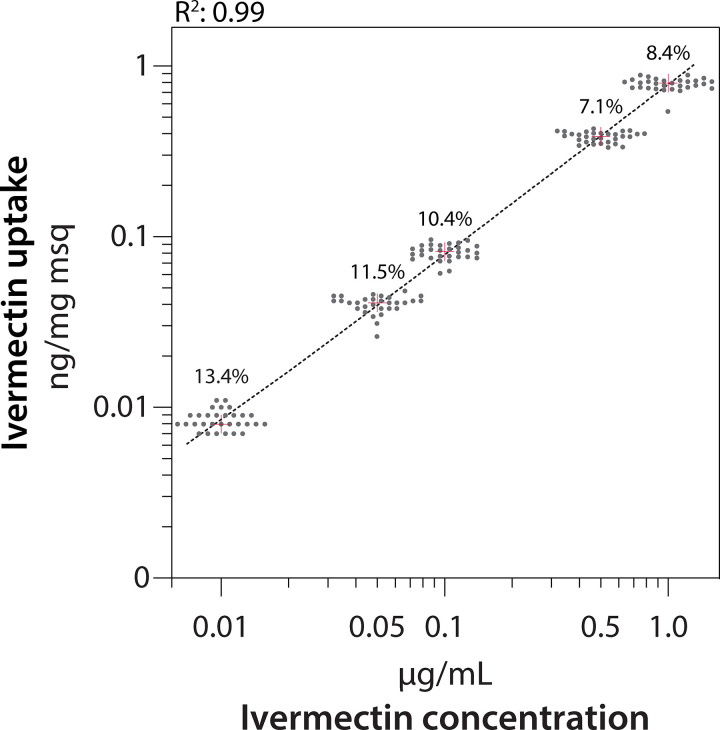
Ivermectin dosing precision and linearity of *Aedes aegypti* mosquitoes. Mosquitoes (n = 30) were treated with blood containing 0.01, 0.05, 0.1, 0.5, and 1 μg/ml ivermectin. The amount of ivermectin shows a linear relationship with the applied concentration in blood (regression line R^2^: 0.99, black dashed line), while the coefficient of variation (CV%) was ≤13.4% (numbers above the regression line). The grey circles correspond to ivermectin amounts recovered from single extracted mosquitoes. The red cross depicts the median amount of ivermectin recovered from the mosquitoes.

The ability to quantify ivermectin in a single mosquito at different time points post treatment and to dose them precisely were important prerequisites for investigating the PK properties of ivermectin in mosquitoes. The dosing precision (%CV) was within 7.1% to 13.4% when adjusted for mosquito weight (Fig 1). The bias was larger (17.7–19.3%) by calculating the ivermectin concentration per individual mosquito, thus the concentration data were henceforth normalised by the body weight.

A linear relationship was found between the dose in the blood meal and the amount recovered from the mosquitoes (R^2^: 0.99). Within the range tested, no saturation was observed, indicating that the mosquitoes neither imbibed less blood at higher ivermectin concentrations nor was the diuresis after blood intake altered by ivermectin intake (Fig 1). Importantly, ivermectin could be quantified over three orders of magnitude in single mosquitoes. Based on these data, a relatively low concentration of 0.1 μg/ml ivermectin could be chosen for PK experiments under which most mosquitoes survived longer than three days.

### Pharmacokinetic studies

#### Pharmacometric analysis of ivermectin

In total, 1108 mosquitoes were analysed by LC-MS/MS; 12 less than planned, because a large percentage of the mosquitoes died 48 h after treatment with ivermectin plus ritonavir. The ivermectin concentration was below the lower limit of quantification in 34 samples, 33 of which were collected 72 h post treatment.

All models were built on the total amounts of substance detected in each mosquito. For the basic structural model, the mosquito was assumed as a single compartment from which the drug disappears. Different types of elimination (zero-order, first-order, Michaelis-Menten) were evaluated while allowing for a lag parameter until the onset of drug elimination. The final basic model was a one-compartment model with zero-order elimination, a lag time parameter, and an estimation of the amount absorbed from the blood meal (as bioavailability F1 from a standard dose).

Covariates considered in the analysis were weight, batch number, and co-administered drugs. Weight was standardized to the median mosquito weight of 3.4 mg and entered as a covariate on dose absorbed and elimination rate (k_el_) with allometric scaling. Inter-occasion variability was observed for the dose administered between different feeding batches. Ritonavir co-treatment was explanatory for the prolonged lag time until elimination in the respective batches. We found no other interaction of the co-administered drugs with the PK of ivermectin in *Ae*. *aegypti*.

**Fig 2:**
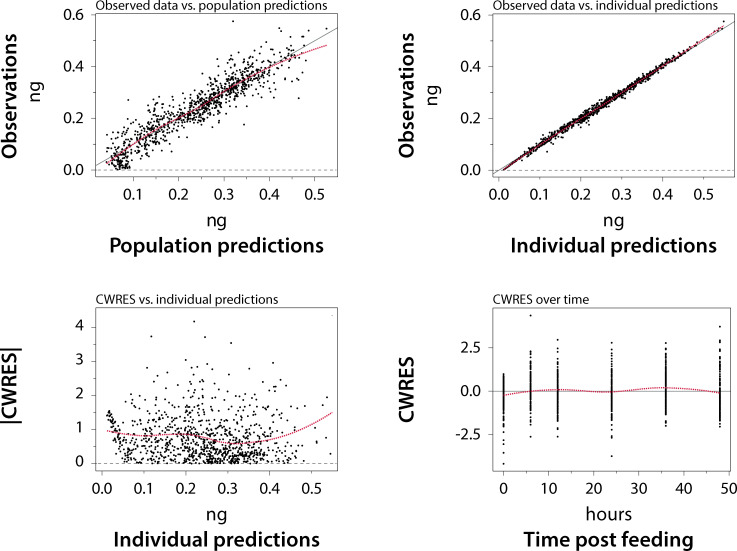
Goodness-of-fit plots for the final model (dashed lines: Local weighted smooth). Observed data vs. population predictions (top left), observed data vs. individual predictions (top right), absolute conditional weighted residuals (CWRES) vs. individual predictions (bottom left), and CWRES over time (bottom right).

Parameter estimates and results of the bootstrap analysis (n = 1000) are shown in Table 1. A total number of 68 runs failed and were excluded from the analysis. Basic goodness-of-fit plots and visual predictive checks by co-administered drug are shown in Figs 2 and S1, respectively. The diagnostic plots show that the observed data are captured well by the final model.

**Table 1:** Final parameter estimates and results of a non-parametric bootstrap analysis (1’000 runs). %RSE: relative standard error (%RSE = 100 x standard error/parameter estimate), CI: confidence interval, k_el_: elimination rate, T_lag_: lag time to elimination, T_lag_add_rit_: additional lag time to elimination in ritonavir co-treatment, IOV: inter-occasion variability.

			Bootstrap analysis (n = 1’000)	
Parameter	Estimate	%RSE	Median	CI 90%
**Fixed effects**				
**dose [ng]**	0.28	0.6	0.28	0.277–0.283
**k**_**el**_ **[ng/h]**	0.0055	5.2	0.055	0.0051–0.0059
**T**_**lag**_ **[h]**	18.5	4.5	18.5	17.0–19.8
**T**_**lag_add_rit**_ **[h]**	11.4	14.0	11.4	8.7–14.2
**Inter-individual variability (ω)**				
**dose**	0.075	41.4	0.075	0.062–0.086
**k**_**el**_	0.25	13.8	0.25	0.20–0.30
**IOV dose**	0.11	6.9	0.11	0.08–0.12
**Residual Errors (σ)**				
**proportional**	0.02	39.7	0.02	0.01–0.03
**additive**	0.018	27.1	0.018	0.009–0.026

#### Concentration-time courses of ivermectin and cytochrome P450 modulators in Aedes aegypti

**Fig 3:**
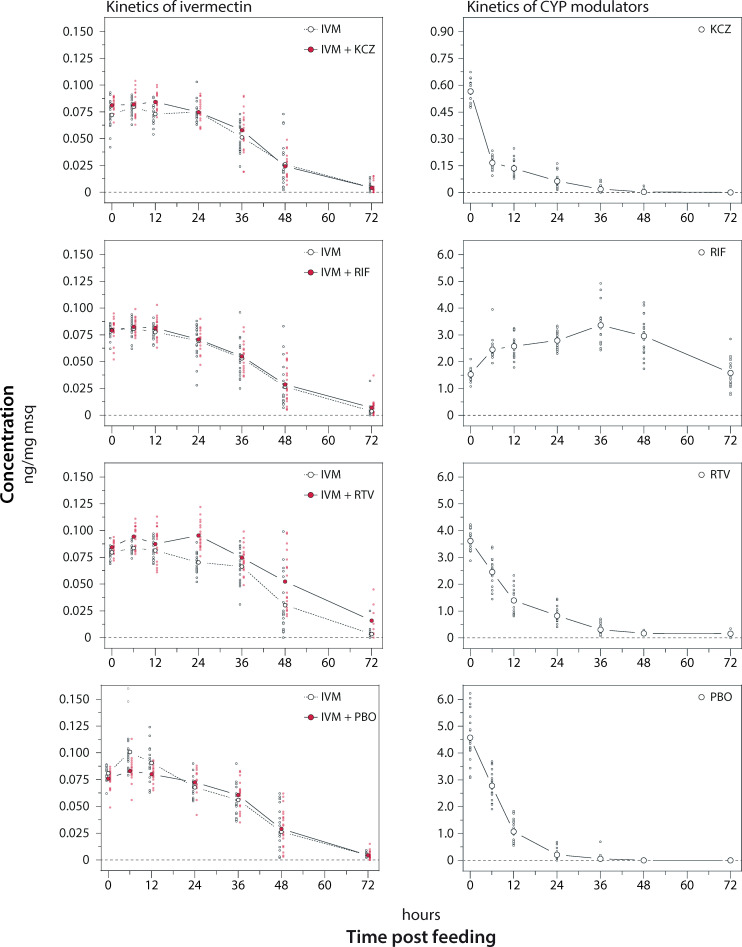
Concentration-time course of ivermectin single and combination treatments in *Aedes aegypti* mosquitoes. *Aedes aegypti* mosquitoes were treated with ivermectin (IVM: 0.1 μg/ml) alone or in combination with ketoconazole (KCZ: 5 μg/ml), rifampicin (RIF:10 μg/ml), ritonavir (RTV: 10 μg/ml), or piperonyl butoxide (PBO: 10 μg/ml). At 0, 6, 12, 24, 48, and 72 h post treatment, the drug concentration in up to 20 live mosquitoes was analysed. Concentration-time profiles of ivermectin single and combination treatments are illustrated in the left column and the profile of the respective CYP modulator in the right column. Large circles depict the mean concentration and the small circles (single treatment: white, combination treatment: red) represent the drug amount extracted per individual mosquito.

Human blood spiked with 0.1 μg/ml ivermectin was administered to four *Ae*. *aegypti* batches, each including at least 140 mosquitoes (Figs 3 and S2). The mosquitoes imbibed on average 0.36 ± 0.06 (SD) ng of ivermectin per feeding, which corresponds to 3.6 μl of blood. The mean weight of a mosquito measured directly after the feeding was 4.5 mg and varied between the mosquito batches between 3.9–5.0 mg (S3 Fig). In the first 18 h after blood-feeding (lag phase) the mosquitoes eliminated excess liquid from the blood meal, with the effect that the concentration of ivermectin remained stable or even increased in the mosquitoes. Following the lag phase, ivermectin was slowly eliminated between 12–24 h post-feeding and after 72 h only traces of ivermectin were still detectable. Overall, the rate of the blood meal digestion was comparable between mosquito batches, resulting in weight loss of 50.7–54.6% over 72 h.

We determined the amounts of CYP450 modulators administered alongside ivermectin to gauge uptake and duration of exposure. Concentration-time profiles are shown in Fig 3. Ketoconazole was eliminated rapidly, with 75% of the dose being excreted already after 6 h, while only few samples could be quantified 36 h post-feeding. No effect on the concentration-time course of ivermectin was observed. In contrast to ketoconazole, rifampicin signals slowly increased in the mosquitoes and reached maximal concentrations approximately after 36 h. Rifampicin was almost not eliminated during the first 2 days, yet the mosquitoes halved their body weight following digestion of the blood meal (S3 Fig). The concentration-time course of ivermectin alone and in combination with rifampicin matched during the entire observation period of 72 h. Thus, rifampicin did not alter the PK of ivermectin. Piperonyl butoxide elimination appeared linear and it was almost completely excreted from the mosquitoes after 36 h. Like the other two CYP modulator, piperonyl butoxide did not change the PK of ivermectin in *Ae*. *aegypti* mosquitoes. Exclusively ritonavir, that appeared to follow first order elimination, influenced the disposition of ivermectin. Ritonavir delayed the start of ivermectin elimination by approximately 11.4 h, resulting in a 29% increased AUC compared to ivermectin alone. Interestingly, the elimination rate of ivermectin was not affected by ritonavir.

### Drug effect of the treatments

The effect of ivermectin, ketoconazole, rifampicin, ritonavir, and piperonyl butoxide on mosquito mortality, fecundity, and fertility was investigated. The results of the drug combinations including ivermectin plus ketoconazole, rifampicin, ritonavir, or piperonyl butoxide were compared with the respective drugs administered alone. In the control groups, mosquitoes received a drug-free blood meal (blank blood).

**Fig 4:**
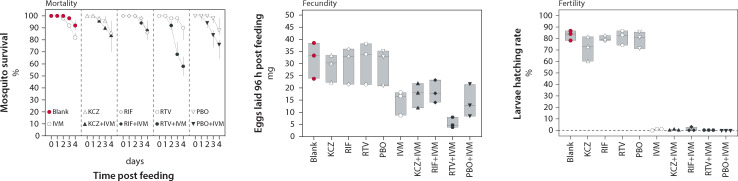
Drug effect of mono- and combination treatments on mosquito mortality, fecundity, and fertility. *Aedes aegypti* mosquitoes were treated with blank human blood, blood containing ivermectin (IVM: 0.1 μg/ml), ketoconazole (KCZ: 5 μg/ml), rifampicin (RIF: 10 μg/ml), ritonavir (RTV: 10 μg/ml) or piperonyl butoxide (PBO: 10 μg/ml), or a drug combination of ivermectin (0.1 μg/ml) plus ketoconazole (5 μg/ml), rifampicin (10 μg/ml), ritonavir (RTV: 10 μg/ml) or piperonyl butoxide (10 μg/ml). 3 batches of 50 mosquitoes were investigated per treatment group. Median percentage survival of the mosquitoes was assessed after 1, 2, 3, and 4 days. The error bars correspond to the range. The fecundity, the amount of eggs excreted, and fertility, the proportion of hatched larvae, were measured after 4 days. The floating-bars display the range and the line in the middle the median value.

After a blank blood meal, 92% of the mosquitoes survived for four days in the climate chamber (Fig 4). Monotherapy with ketoconazole, rifampicin, ritonavir, and piperonyl butoxide resulted in four-day mosquito mortality of 10–13%, whereas ivermectin killed 17% of the mosquitoes in the first four days. The four-day mosquito mortality of ivermectin plus ketoconazole, rifampicin, and piperonyl butoxide was 19%, 12%, and 27%, respectively, suggesting no additive effect. In contrast, the combination of ivermectin and ritonavir killed 42% of the mosquitoes in four days, which was more than the respective single drug treatments (p-value < 0.001 compared to treatments with blank blood, or ivermectin alone). Ivermectin was the only drug that reduced the number of mosquito eggs compared to blank blood feedings (p-value < 0.023 compared to treatments with blank blood). This effect was even stronger when ivermectin was combined with ritonavir (p-value < 0.001) or PBO (p-value < 0.02) compared to blank blood treatments.

After feeding the mosquitoes blank blood, 83% (CI_95%_: 72%-94%) of the eggs were fertile and produced live larvae. The CYP modulators did not reduce the fertility of the mosquitoes considering that 72% to 81% of the eggs produced live larvae. Ivermectin alone sterilised almost all mosquito eggs (p-value < 0.04 compared to blank blood treatments) and, therefore, no increased effect would be observable for ivermectin combinations even if there were an effect.

Overall, when exposed to the sub lethal concentrations of ivermectin used in this study, mosquitoes were almost infertile, while their survival was notably reduced by the co-medication with ritonavir.

## Discussion

Mass administration of endectocidal drugs might be a valuable addition to insecticide-treated bed nets and indoor residual spraying because it offers the opportunity to also target mosquitoes that bite outdoors or have diurnal activity. However, co-medications may impact the effectiveness of mass drug administration campaigns by changing drug disposition not only in treated humans but also in the mosquito. PK investigations in mosquitoes are, therefore, not only crucial for investigating antagonistic or synergistic drug-drug interactions, but also for optimising lead compounds and determine species- or population-specific differences. Our study demonstrates that the PK of small molecules can be assessed in blood-feeding mosquitoes and reveals that each investigated compound exhibited unique kinetic characteristics. Furthermore, we showed that the disposition of ivermectin can be modulated by co-treatment of ritonavir, thereby increasing the mosquitocidal activity.

The first prerequisite for realising PK investigations in mosquitoes is a consistent volume of the blood ingested across individuals to allow for precise dosing. Ivermectin concentrations and body weight as determined directly after the mosquitoes had fed from the membrane feeder varied by less than 13.4% and 15%, respectively, showing that the blood intake of *Ae*. *aegypti* mosquitoes was very uniform. The second prerequisite is that the drug in the meal does not affect blood intake. Overall, the mosquitoes ingested a consistent volume of blood, regardless of the ivermectin concentration (0.01–1 μg/ml), considering that the mosquito weights were very similar across the different treatment dosages (range: 3.0–3.3 mg). Moreover, a strong linear relationship was observed between the administered ivermectin blood concentration and the amount measured in the mosquitoes (Fig 1). Conveniently, also high ivermectin dosages did not exert a repelling effect on *Ae*. *aegypti* mosquitoes, and the extraction recovery of the bioanalytical method was reproducible over at least two orders of magnitude without saturation. Finally, the method sensitivity was sufficient to quantify 98.5% of the elimination process of ivermectin in single mosquitoes, demonstrating that this setup allows for measuring the PK over a large dynamic range.

The mosquitoes ingested on average 3.6 μl blood, based on the amount of ivermectin measured after the feeding. This is on par with previous studies that found the volume of a regular blood meal ranging between 2.4 to 4.5 μl [20–22]. The weight loss over 72 h was on average 2.4 mg, corresponding to about 2.3 μl blood (δ_Blood_: 1.06), thus approximately 1.4 mg less than expected, assuming that blood digestion was complete. However, the digestion process was most likely not terminated at this time point because, according to Redington and Hockmeyer (1976), it takes around four days to completely excrete radiolabelled blood [22]. Furthermore, the blood uptake is underestimated by gravimetric measurements given that approximately 20–30% of the total imbibed fluid is excreted by the mosquitoes during and for up to 3 h after the feeding [22,23]. It was striking that immediately after feeding (T_0_), especially ketoconazole and rifampicin concentrations were considerably lower than what would be expected from the amount of blood taken up by the mosquitoes. The mosquitoes were allowed to feed for 20 min on blood while they were already excreting large amounts of liquid during diuresis, which possibly contained significant quantities of the drugs. Another potential reason for the observed discrepancy could be that the drugs were rapidly metabolised during the feeding in the insect’s midgut. Lastly, it cannot be excluded that ketoconazole and rifampicin were incompletely recovered by the employed extraction method.

To the best of our knowledge, this is the first drug-drug interaction study using arthropods as model organisms, and the first to determine the PK properties of ivermectin in mosquitoes. Studies in ruminants and humans have shown that the highly lipophilic ivermectin binds strongly to plasma proteins, distributes widely in the body, while accumulating in fatty tissue, leading to a prolonged elimination [24,25]. In *Ae*. *aegypti*, ivermectin accumulates in the mosquitoes during an initial lag phase of around 18 h before being slowly eliminated over a period of 2 days. In contrast to the exponential decay observed in mammalian species, ivermectin elimination in mosquitoes followed zero-order kinetics. The concentration-time profile of rifampicin resembled the one of ivermectin, whereas the amplitude of the rifampicin curve was greater. Ketoconazole, piperonyl butoxide and ritonavir, on the other hand, followed a first order elimination similar to observations made with radio labelled ^14^C-permethrin, which was applied topically on the sternum or notum of *Ae*. *aegypti* mosquitoes. There, permethrin was transformed by CYPs to hydroxy-permethrin and other more hydrophilic metabolites [26]. Involvement of CYP metabolism in *Ae*. *aegypti* is also conceivable for piperonyl butoxide as it is known to be a substrate of mammal as well as insect CYPs [19].

Ritonavir was the only CYP modulator, which significantly increased ivermectin levels in the mosquitoes. It is a broad-spectrum inhibitor of human CYPs, UGTs, and of the efflux pump P-glycoprotein and other drug transporters [27]. Ritonavir prolonged the lag-time of ivermectin by about 11 hours but not the elimination rate, which might be an indication that an efflux pump rather than the function of metabolic enzymes was inhibited. This is plausible as the *Ae*. *aegypti* genome consists of numerous putative adenosine triphosphate binding cassette (ABC) transporters including multidrug efflux proteins [28]. Considering that piperonyl butoxide did not influence the PK of ivermectin, even though many studies demonstrated that it is a potent and non-selective inhibitor of CYPs in mammals and invertebrates, supports this hypothesis [29]. However, it cannot be excluded that its inhibitory capacity was hindered due to the lower bioavailability of the oral compared to topical route of application. Interestingly, Chaccour and colleagues observed that ketoconazole increased the exposure of ivermectin in pigs as well as the activity of ivermectin against *An*. *gambiae* mosquitoes possibly by inhibiting CYPs in both organisms [18]. However, the exposure and efficacy of ivermectin in *Ae*. *aegypti* was not altered by ketoconazole co-treatment, potentially because *Ae*. *aegypti* possesses a larger pool of detoxification genes than *An*. *gambiae*, leading to rapid inactivation of ketoconazole [30]. Fast elimination was observed in *Ae*. *aegypti*, however the pharmacokinetic interaction of ivermectin and ketoconazole must be investigated in *Anopheles* mosquitoes to understand those species differences. Major classes of inducers of vertebrate CYPs such as barbiturates and alkaloids are also functional in insects [31]. Nevertheless, rifampicin, which is a potent inducer of numerous CYPs, members of the uridine 5’-diphospho-glucuronosyltransferases (UGT), and the efflux transporter P-glycoprotein in humans [32], did not accelerate elimination of ivermectin in *Ae*. *aegypti*. This result must be interpreted with caution, because the increase of the protein pool is not immediate, requiring usually pre-exposure to the inducer over several days [32–34]. Given that in the wild exposure to rifampicin would occur simultaneously with ivermectin, our study allows for a realistic estimate of the impact of this interaction. Overall, quantification of ivermectin metabolites and analysis of the mosquito excrements would have been prerequisite to elucidate the mechanism of drug interactions in *Ae*. *aegypti*.

Ivermectin is only active against *Aedes* mosquitoes at blood concentrations that cannot be obtained in humans receiving a regular dose [13,35]. Studying the pharmacokinetics of ivermectin in *Aedes* spp. has the advantage that higher dosages can be administered, which facilitates sampling of live mosquitoes and drug quantification in single specimens. Nonetheless, the use of *Aedes* as model to infer information that pertains to ivermectin as endectocide remains unclear and necessitates comparative studies in highly susceptible mosquito species such as *An*. *gambiae* [36]. Importantly, the pharmacokinetic interaction between ritonavir and ivermectin translated into a more pronounced effect considering that the mosquito mortality increased, and the number of eggs laid decreased. Ivermectin reduced the fertility and fecundity of *Ae*. *aegypti* mosquitoes, which is in line with previous observations [37–39]. However, the effect on the hatching rate was more pronounced than reported by Focks and Hadlett [35,40], arguably because the susceptibility varies between *Ae*. *aegypti* strains [38]. The effect on the ovarian development should not be overrated given that the reduced fecundity and fertility ends when ivermectin is discontinued [37].

In conclusion, this study demonstrates for the first time that the PK of ivermectin can be measured in mosquitoes upon blood feeding. The kinetic properties of ivermectin can be modulated by co-administered drugs leading to increased mosquitocidal activity. The current method will advance our understanding of endectocidal pharmacology in mosquitoes and may help in designing novel or improving existing compounds for mosquito control.

## Materials and methods

### Chemicals, reagents and reference substances

Ammonium format and dimethyl sulfoxide (DMSO) were purchased from Sigma-Aldrich (Buchs, Switzerland). Gradient grade methanol, water, formic acid (FoA, 98–100%), isopropyl alcohol, and acetonitrile were products of Merck (Darmstadt, Germany). Human blank blood, stabilised by citrate-phosphate-derivative with adenine, was obtained from the local blood donation centre.

Ivermectin and ritonavir as well as the internal standard ivermectin-d_2_ were purchased from Toronto Research Chemicals (Toronto, Canada). Rifampicin, ketoconazole, and piperonyl butoxide were obtained from Sigma-Aldrich.

### Mosquito rearing

*Ae*. *aegypti* Rockefeller strain were reared under constant temperature (27° C ± 2° C) and humidity (70% ± 10%) conditions, and at a 12:12 hours light dark cycle. Female and male mosquitoes were kept in the same cage to allow for mating to occur. Females were membrane fed once a week with fresh pig blood received from the local abattoir. Eggs were harvested on round filter papers (⌀: 8 cm, Sartorius, Göttingen, Germany) placed on top of a moist sponge that was kept in a glass bowl. The eggs were then left to sclerotize and dry for seven days in the insectary. The eggs were hatched by placing a slice of the egg-coated filter paper in a glass dish filled with tap water treated with 0.012% (v/v) AquaSafe (Aquasafe, Seevetal, Germany). About 300 larvae were transferred into white plastic trays containing 600 ml AquaSafe-treated water. The larvae were cultivated in an incubator (Aqualytic, Dortmund, Germany) set at 27° C and fed with TetraMin fish food (Tetra, Melle, Germany) on a daily basis. Once the larvae reached the 4^th^ instar they were transferred into emergence cages (375 mm x 445 x mm 495 mm) and adults were provided with 10% aqueous sucrose solution ad libitum.

### Pharmacokinetic studies in *Aedes aegypti* mosquitoes

#### Mosquito treatments

Drug stock solutions were prepared in DMSO and added to blank human blood at a ratio of 1:1000 so that the DMSO content was less than 0.2%. The mixtures were rotated at room temperature for 30 min to ensure a uniform drug distribution (Rotator Genie, Scientific Industries, Bohemia, USA). The sugar water within the mosquito cages was replaced by tap water 24 h prior to the PK experiments. Twelve hours later, the tap water was also removed to starve the mosquitoes. On the day of the PK experiment, 25–35 starved, female mosquitoes with an age of 6–7 days post-emergence were transferred with a mouth aspirator into 2 dl paper cups that were sealed with a piece of mosquito netting. The blood samples were heated in a water bath (SAHARA PPO S5P Heated Bath Circulators, Thermo Fisher Scientific, New Hampshire, USA) for 20 min at 39.5°C. A petri dish (35 mm x 10 mm, BD, Franklin Lakes, USA) was filled with 2.55 ml of warm blood, sealed with a piece of Parafilm (Parafilm M sealing film, Huberlab AG, Aesch, Switzerland) stretched over the rim and turned upside down on top of the paper cup. The mosquitoes were then feeding from blood through the netting and the Parafilm membrane. The feeding was conducted in a climate chamber (HPP110, Memmert GmbH + Co.KG, Schwabach, Germany) at 26° C, 70% humidity, and 50% light intensity. The photoperiod of the climate chamber and the insectary were synchronised. After 20 min of feeding, the cups were placed on ice to immobilise the mosquitoes and separate the fully engorged ones. The engorged mosquitoes were either directly frozen at -20°C or returned to the climate chamber for certain time periods to assess the PK.

#### Mosquito dosing experiments

The first aim was to evaluate the variability of the blood uptake by the mosquitoes. Batches of 30 *Ae*. *aegypti* mosquitoes were fed on human blank blood containing either 0.01, 0.05, 0.1, 0.5 or 1 μg/ml ivermectin. The dosing precision was estimated by the coefficient of variation (CV%), i.e. the ratio of the standard deviation to the mean (Excel Office 365, Microsoft, Washington, USA). The second aim was to measure the association between the ivermectin concentration in the blood and the amount recovered from the mosquitoes. The strength of the association was assessed using a linear regression model and its correlation coefficient R^2^ using GraphPad prism software 9.0.0 (San Diego, USA). On the basis of these results an optimal dose for the PK experiments was chosen yielding a strong ivermectin baseline signal while keeping its concentration at a level sub-lethal for the mosquitoes.

#### Pharmacokinetic studies

Based on the dosing experiments, female mosquitoes were membrane fed human blood containing 0.1 μg/ml ivermectin alone or a combination of ivermectin (0.1 μg/mL) with one of the following CYP modulators: ritonavir (10 μg/ml), ketoconazole (5 μg/ml), rifampicin (10 μg/ml), or PBO (10 μg/ml). The treatments were carried-out on four different occasions (four batches of mosquitoes), in which ivermectin was always administered alone as well as in combination with one of the four CYP modulators. Consequently, the effect of the combination therapy could be compared one-to-one with the monotherapy, thereby preventing variations caused by different mosquito batches. After feeding, fully engorged mosquitoes were frozen at -20° C (T_0_) or returned into the climate chamber for an incubation period of 6, 12, 24, 36, 48, or 72 h post feeding (T_6-72h_). At each time point, 20 live mosquitoes of each treatment condition were frozen at -20° C until sample analysis. Concentration-time courses were plotted using GraphPad Prism 9.0.0. Mean values and standard deviations (S2 Fig) or each single value (Fig 3) were illustrated.

#### Bioanalysis of the mosquito samples

Each mosquito was weighed on a precision balance (Mettler Toledo, XP26 Excellence Plus, Ohio, USA) and placed into a 1.5 ml microtube that contained 100 μl of the extraction solvent, a mixture of methanol:water (7,3 v/v) containing a final concentration of 0.1% formic acid. The tissue was crushed with a cordless motor-driven tissue grinder using disposable pellet pestles (DWK Life Sciences, Wertheim, Germany). The grinding was performed until the mosquitoes were completely disintegrated. Afterwards, an additional volume of 75 μl extraction solvent was added to the crushed mosquito samples. The samples were mixed for 30 seconds in a multi tube vortex mixer (Marshall Scientific, Hampton, USA), sonicated for 10 min (Bandelin Sonorex ultrasonic bath, Faust, Schaffhausen, Switzerland), and centrifuged for 10 min at 16.1 x g at room temperature (5415R micro-centrifuge, Eppendorf, Hamburg, Germany). In a next step, 135 μl of the supernatant was transferred into autosampler tubes and mixed with 15 μl internal standard solution (IS). The IS contained 125 ng/ml ivermectin-d_2_ and was prepared in a 1:1 (v/v) methanol:water mixture supplemented with formic acid to a final concentration of 0.1%. Finally, the autosampler tubes were centrifuged (5810R centrifuge, Eppendorf) for 30 min at 15°C and 3220 x g and placed into the autosampler (10°C) for subsequent liquid chromatography tandem mass spectrometry (LC-MS/MS) analysis.

Mosquito extracts were analysed using a modular ultra-high-pressure liquid chromatography (UPLC) system from Shimadzu (Kyoto, Japan) connected to an API 5500 QTRAP tandem mass spectrometer (AB Sciex, Massachusetts, USA). A previously developed and validated LC-MS/MS method for the quantification of ivermectin in human plasma and blood was used for the analyses [41]. The method was slightly adapted in order to analyse ritonavir, ketoconazole, rifampicin, and piperonyl butoxide next to ivermectin. The compound specific settings for all analytes are listed in S1 Table.

Reference stock solutions for calibration and quality control (QC) samples were prepared in DMSO at a concentration of 1 mg/ml for ivermectin and 10 mg/ml for ritonavir, ketoconazole, rifampicin, and piperonyl butoxide. The reference solutions were mixed with methanol:water (1:1, v/v) in a 1:100 (v/v) ratio in order to obtain a calibration and a QC working solution mixture. These mixtures were then further diluted with blank mosquito matrix to a final concentration of 0.1 μg/ml ivermectin and 1 μg/ml for the other four analytes. A blank mosquito matrix was produced by feeding mosquitoes drug-free human blood while following the same extraction protocol as described above. Calibration and QC samples were prepared afterwards by serial dilution in pooled blank mosquito extracts. The calibration ranges were 0.025–50 ng/ml for ivermectin, 0.25–100 ng/ml for ketoconazole, 0.25–500 ng/ml for rifampicin, 0.25–1000 ng/ml for ritonavir, and 2.5–500 ng/ml for piperonyl butoxide. Each calibration set consisted of one double blank (only matrix), a blank (matrix and IS) as well as up to 11 calibrators. QC samples were prepared at low, mid, and high concentration so as to cover the entire calibration range. For ivermectin QC samples with 0.1, 1, and 10 ng/ml were prepared, while the concentrations were ten times higher (1, 10, and 100 ng/ml) for ketoconazole, rifampicin, ritonavir, and piperonyl butoxide.

Aliquots of 135 μl were transferred into autosampler tubes and stored at -20°C. For each PK analysis a set of calibration and QC samples were thawed, processed as for the PK samples, and analysed at the beginning, middle, and end of the analytical run. The PK samples were quantified with the Multi Quant software 3.0.3 (AB Sciex). The calibration points were fitted by a linear regression model (weighted 1/x^2^) of the analyte concentration (x) against the peak area ratio of the analyte and the IS (y). No IS was used for ketoconazole, rifampicin, ritonavir, and piperonyl butoxide.

The correlation coefficient R of the calibration lines was in each analytical run larger than 0.997. The intra-run precision of ivermectin QC samples was ≤8% (n = 3), while the inter-run precision was ≤6% (n = 12). The intra- and inter-run accuracy of ivermectin was between 99.8% to 107.2%, and 103.6% to 104.6%, respectively. The accuracy of the CYP modulator QC samples was between 93.6% to 109.4% and the precision was ≤15%. These data indicate that the bioanalytical method was reliable for the quantification of ivermectin as well as for the CYP modulators in single mosquitoes.

#### Pharmacometric analysis

All analyses were performed on whole-body amounts of drug in individual mosquitoes, i.e. the amount of drug detected in each mosquito specimen. As the dose administered can vary depending on the feeding behaviour of individual mosquitoes, the amount of drug taken up during the blood meal was estimated as an additional model parameter (bioavailability of a standard dose, F1).

Data examination, analysis, and visualization were performed in GNU R (Version 3.5.3; R Foundation for Statistical Computing, http://www.R-project.org, Vienna, Austria). Population pharmacokinetic analysis was done using NONMEM (Version 7.4.3; Icon Development Solutions, http://www.iconplc.com, Ellicott City, MD, USA) with the help of the Xpose and PsN software packages for model diagnostics and testing of covariate relationships [42,43]. The first order conditional estimation with eta-epsilon interaction (FOCE-I) was used throughout the entire process.

For covariate model building, first a visual examination was done and covariates were selected by clinical plausibility, after which the effect of weight (the only covariate available) on structural model parameters was tested. Models were selected based on goodness-of-fit statistics (objective function value (minus twice the log-likelihood)), graphical analysis using visual predictive checks (VPCs, n = 1,000 simulations), and biological plausibility. Non-parametric bootstrap analysis (n = 1,000) was used to assess the precision of the estimates in the final model.

### Drug effect of the treatments

The drug effect on mosquito survival, fecundity and fertility was assessed over an incubation period of four days in three replicates of 50 blood-fed mosquitoes for each condition; blank human blood (negative control), blood with ivermectin (0.1 μg/mL), ritonavir (10 μg/mL), ketoconazole (5 μg/mL), rifampicin (10 μg/mL), or PBO (10 μg/mL). In addition, drug combinations of ivermectin (0.1 μg/ml) with either ritonavir (10 μg/ml), ketoconazole (5 μg/ml), rifampicin (10 μg/ml), or piperonyl butoxide (10 μg/ml) were tested. Blood feeding was done as described above. After the feeding, fully engorged mosquitoes were divided into groups of 25 per paper cup and placed in the climate chamber for 96 h. In contrast to the PK experiments, the mosquitoes had unlimited access to water because a moistened oviposition substrate was inserted into the paper cup.

The drug effect on mosquito survival was assessed by counting the living mosquitoes at 24, 48, 72 and 96 h post-treatment. Mosquitoes still moving upon physical contact were considered being alive.

Fecundity was defined as the total number of eggs laid after 96 h. The eggs were collected inside the paper cups with a small petri dish (35 mm x 10 mm) that was padded with water-soaked cotton wool and covered with filter paper (⌀: 42.5 mm, Whatman, Maidstone, United Kingdom). The filter paper served as the oviposition substrate. The cotton wool was soaked with fresh water and covered by a new filter paper after 48, 72, and 96 h post-treatment. After the assay, the eggs were left to mature for 24 h in the insectary. The eggs were brushed carefully from the filter paper into a new petri dish (35 mm x 10 mm) and weighed on a precision balance (Mettler Toledo, XP26 Excellence Plus, Ohio, USA) to estimate the amount of eggs laid per treatment.

In order to evaluate the fertility of the mosquitoes, 100 eggs that were laid between 48 h and 72 h (n = 50) and between 72 h and 96 h (n = 50) post-treatment were immersed in 40 ml tap water supplemented with 0.012% AquaSafe for 48 h. Any hatched larva was counted and transferred to a rearing tray with a plastic pipette. The fertility was calculated as the percentage of eggs hatched.

Drug effects on mortality, fecundity, and fertility were performed in GNU R (Version 3.5.3; R Foundation for Statistical Computing, http://www.R-project.org, Vienna, Austria). Normality was assessed using the Shapiro-Wilk test. For normally distributed data (mortality, fecundity), group differences were assessed with one-way ANOVA testing, and, where applicable, followed up with Tukey’s test. Group differences in fertility (not normally distributed) were evaluated with Kruskal-Wallis one-way analysis of variance, and Wilcoxon rank sum testing as post-hoc. P-values are reported with adjustment for multiple comparison and were considered significant if p-value < 0.05.

## Supporting information

S1 Fig Visual predictive check (VPC). (PDF)

S2 Fig Concentration-time course of ivermectin determined in four different *Aedes aegypti* mosquito batches. (PDF)

S3 Fig *Aedes aegypti* weight progression over 72 h after feeding on human blood. (PDF)

S1 Table Compound specific mass spectrometry settings and calibration and quality control samples used for drug quantification. (PDF)

S1 Data Raw data of figures. (XLSX)
