# Supplementary material for: The pharmacokinetics and drug-drug interactions of ivermectin in Aedes aegypti mosquitoes
Source: PLoS Pathog. 2021 Mar 17;17(3):e1009382. doi: 10.1371/journal.ppat.1009382 (PMC7968666; doi:10.1371/journal.ppat.1009382)
Supplement: S1 Fig — (PDF) [file ppat.1009382.s001.pdf]

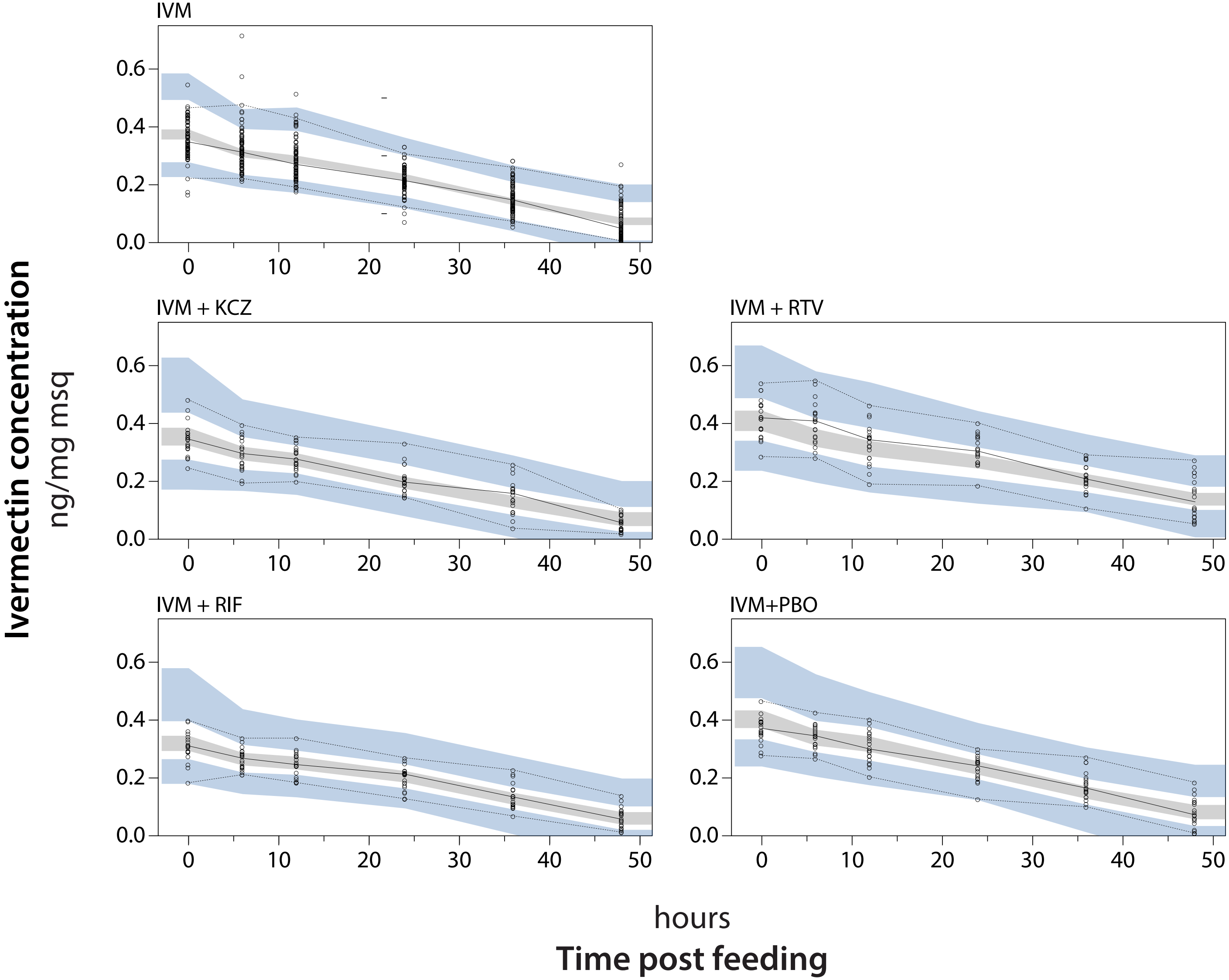

Figure S1. Visual predictive check (VPC).

VPC (n=500 simulations) with 90% CI bands (5th and 95th percentiles shaded blue, median shaded grey) and observed data (dashed lines, 5th and 95th percentiles; solid line, median). IVM: ivermectin, KCZ: ketoconazole, RIF: rifampicin, RTV: ritonavir, PBO: piperonyl butoxide.
