# Supplementary material for: The pharmacokinetics and drug-drug interactions of ivermectin in Aedes aegypti mosquitoes
Source: PLoS Pathog. 2021 Mar 17;17(3):e1009382. doi: 10.1371/journal.ppat.1009382 (PMC7968666; doi:10.1371/journal.ppat.1009382)
Supplement: S2 Fig — (PDF) [file ppat.1009382.s002.pdf]

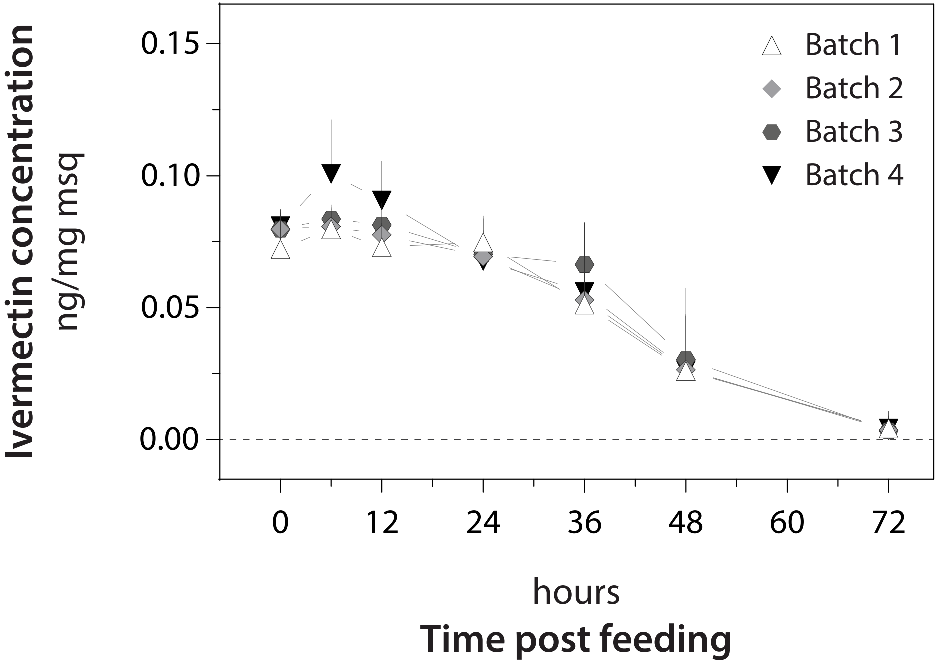

Figure S2. Concentration-time course of ivermectin determined in four different *Aedes aegypti* mosquito batches.

Mosquitoes were collected at multiple time-points (0, 6, 12, 24, 36, 48, and 72h) post-feeding ivermectin (0.1 µg/ml). Symbols correspond to the mean value and error bars to the standard deviation.
