# Supplementary material for: The pharmacokinetics and drug-drug interactions of ivermectin in Aedes aegypti mosquitoes
Source: PLoS Pathog. 2021 Mar 17;17(3):e1009382. doi: 10.1371/journal.ppat.1009382 (PMC7968666; doi:10.1371/journal.ppat.1009382)
Supplement: S3 Fig — (PDF) [file ppat.1009382.s003.pdf]

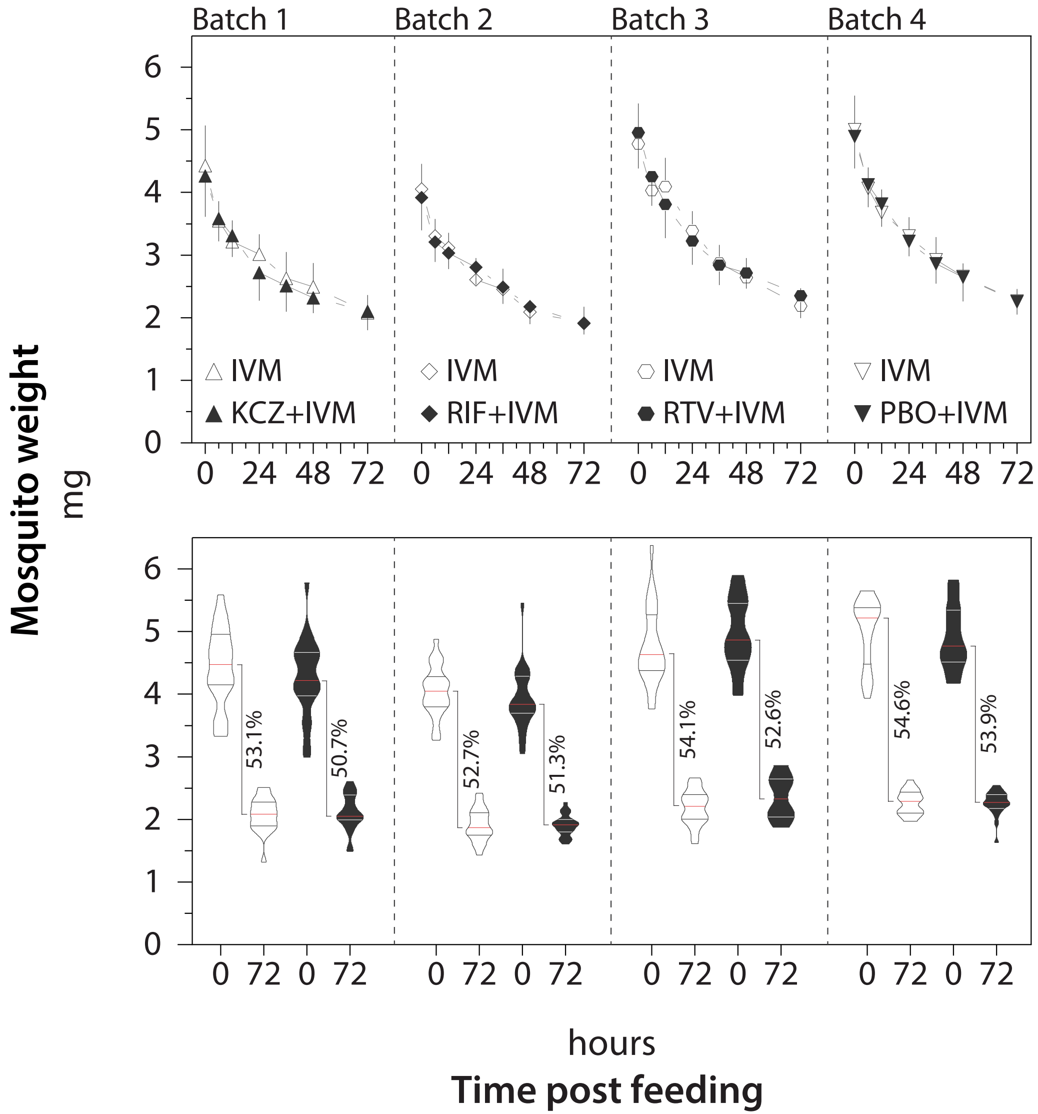

Figure S3. *Aedes aegypti* weight progression over 72 h after feeding on human blood.

Each mosquito batch (1-4) received human blood containing ivermectin (0.1 µg/ml IVM) and a combination of ivermectin (0.1 µg/ml) plus either ketoconazole (batch 1: 5 µg/ml KCZ), rifampicin (batch 2: 10 µg/ml RIF), ritonavir (batch 3: 10 µg/ml RTV), or piperonyl butoxide (batch 4: 10 µg/ml, PBO). The upper graph illustrates the weight progression of each treatment group. White symbols depict the mean weight of ivermectin single treatments, while dark grey symbols represent the mean weight of the combination treatments. The error bars correspond to the standard deviation. The lower graph depicts violin plots of the mosquito weight distribution directly after feeding and after 72 h incubation. Single drug treatments are illustrated in white and combination treatments in dark grey. The red line corresponds to the median value and the area among the upper and lower line to the interquartile range. The numbers indicate the weight loss of the mosquitoes over 72 h.

The mean weight of a mosquito measured directly after the feeding was 4.5 mg, ranging between 3.0-6.4 mg. The initial weight of the different mosquito batches varied in average between 3.9 mg to 5.0 mg, whereas the mosquito weight post-feeding and the weight progression was very similar within the same batch. Seventy-two hours post-feeding, the mosquitoes weighed on average 2.1 mg (range: 1.3-2.9 mg). The weight loss was very consistent between the different mosquito batches considering that the reduction over 72 h was between 50.7% and 54.6%. Overall, the amount of imbibed blood varied between the mosquito batches, yet the rate of the blood meal digestion was comparable.
