# Supplementary material for: The pharmacokinetics and drug-drug interactions of ivermectin in Aedes aegypti mosquitoes
Source: PLoS Pathog. 2021 Mar 17;17(3):e1009382. doi: 10.1371/journal.ppat.1009382 (PMC7968666; doi:10.1371/journal.ppat.1009382)
Supplement: S1 Table — (PDF) [file ppat.1009382.s004.pdf]

Table S1. Compound specific mass spectrometry settings and calibration and quality control samples used for drug quantification. The mass transitions I and II of ivermectin (892.3 → 569.1, 307.2 m/z) as well as of ivermectin-d_2 (895.4 → 571.8, 309.3 m/z) were summed to increase the signal intensity.

Analyte	Q1 m/z	Q3 m/z	DP V	EP V	CE V	CXP V	Retention time min	Calibration range ng/mL	Quality control samples ng/mL	Internal standard
Ivermectin I	892.3	569.1	126	10	21	34	1.89	0.025 - 50	0.1, 1, 10	Ivermectin-d_2
Ivermectin II	892.3	307.2	126	10	33	18	1.89			
Ketoconazole	531.1	489.1	181	10	30	34	1.23	0.25 - 100	1, 10, 100	
Rifampicin	823.3	791.3	81	10	23	54	1.24	0.25 - 500	1, 10, 100	
Ritonavir	721.2	296.2	156	10	13	20	1.37	0.25 - 1000	1, 10, 100	
Piperonyl butoxide	356.3	177.2	106	10	55	12	1.49	2.5 - 500	1, 10, 100	
Ivermectin-d_2 I	895.4	571.8	126	10	21	38	1.90			
Ivermectin-d_2 II	895.4	309.3	126	10	33	20	1.90			
